# Dexmedetomidine on the interplay of IL-6 and STAT3 pathways in adrenal gland damage-induced scalding burns in rats

**DOI:** 10.1007/s00210-024-03300-7

**Published:** 2024-07-23

**Authors:** Serpil Ciftel, Filiz Mercantepe, Tolga Mercantepe, Enver Ciftel, Aleksandra Klisic

**Affiliations:** 1 Department of Endocrinology and Metabolism, Erzurum Regional Training and Research Hospital, Erzurum, Turkey; 2 Department of Endocrinology and Metabolism, Faculty of Medicine, Recep Tayyip Erdogan University, Rize, 53010 Turkey; 3 Department of Histology and Embryology, Faculty of Medicine, Recep Tayyip Erdogan University, Rize, Turkey; 4 Department of Endocrinology and Metabolism, Sivas Numune Hospital, Sivas, Turkey; 5 University of Montenegro-Faculty of Medicine, Podgorica, Montenegro; 6 Center for Laboratory Diagnostics, Primary Health Care Center, Podgorica, Montenegro

**Keywords:** Adrenal gland, Burn, Dexmedetomidine, IL-6, Rat, STAT3

## Abstract

Scalding burns are a common form of thermal injury that often leads to systemic complications. Pro-inflammatory cytokines like interleukin-6 (IL-6) and the activation of signal transducer and activator of transcription 3 (STAT3) pathways have been linked to the pathophysiology of organ damage caused by burns. This study aimed to investigate the potential therapeutic effects of dexmedetomidine, an α2-adrenergic receptor agonist with anti-inflammatory properties, on the interplay of IL-6 and STAT3 pathways in adrenal gland damage following scalding burns in rats. Twenty-eight rats were divided randomly into four groups. Rats in group 1 (*n*=7, control) were given only 0.9% intraperitoneal (i.p.) NaCl. Rats in group 2 (*n*=7, DEX) were exposed to 25°C water for 17 s on day 1 and received 100 mcg/kg/day dexmedetomidine i.p. for 3 days; for rats in group 3 (*n*=7, Burn), boiling water of 94°C was applied inside for 17 s. Rats in group 4 (*n*=7, Burn+DEX) were exposed to 94°C water for 17 s and received 100 mcg/kg/day dexmedetomidine i.p. for 3 days. Adrenal gland tissues were histopathological examined, and STAT3, IL-6, and TUNEL staining were performed using immunohistochemically. Our results revealed that scalding burns increased IL-6 and STAT3 expression in the adrenal glands of rats. Histological analysis demonstrated that dexmedetomidine administration ameliorated adrenal gland damage and reduced inflammatory cell infiltration. Our findings suggest that dexmedetomidine protects the adrenal glands in scalding burns. This protection appears to be mediated, at least in part, by its modulation of IL-6 and STAT3 pathways.

## Introduction

Burn injuries are unfortunate events with significant physical, psychological, and economic effects on the individual and society. Burn injuries represent a common problem with notable global differences (Keck et al. 2009). According to the literature, burn-related complications are estimated to cause 180 thousand deaths per annum, which renders these injuries an essential public health problem (American Burn Association 2019). Understanding the consequences of burn injuries is very important for the planning of treatment and health services. Burn injuries occur due to various causes including fire burns, scald burns, electrical burns, and chemical burns (AbuBakr et al. 2018). Especially in young children and older adults, scalding is caused by hot liquids or steam (Plancq et al. 2016). Occupational hazards, domestic accidents, and deliberate actions also contribute to the causes of burn injuries.

The severity of burn injuries varies and is usually classified based on the depth and extent of tissue damage. The literature reveals that a significant portion of burn injuries are classified as mild or moderate severity, which includes partial thickness burns (AbuBakr et al. 2018). However, severe burns that also include full-thickness burns may lead to long-term disability and deformation. Burn injuries represent a multifaceted problem that extends beyond visible skin damage. The literature emphasizes that burn injuries may frequently trigger a series of systemic responses, including inflammation, oxidative stress, and changes in immune function (Plancq et al. 2016; AbuBakr et al. 2018). The severity and extent of these responses depend on factors such as burn depth, the total body surface area that has been affected, and the patient’s overall health (Kotzbeck et al. 2019).

Severe burns cause sudden and deep tissue damage at the injury site (Ladhani et al. 2021). The initial effect of a burn injury is localized destruction of skin layers and the tissues underneath. Burn severity, which is measured based on factors such as burn depth and surface area, determines the extent of tissue damage (Laggner et al. 2022). This local injury triggers a series of events that extend beyond the initial site and affect distant organs (Burgess et al. 2022). After a severe burn, an intense inflammatory response is initiated by the mediation of the body’s defense mechanism (Costantini et al. 2022). This response involves the release of various proinflammatory cytokines and mediators such as tumor necrosis factor-alpha (TNF-α) and interleukins (Keck et al. 2009). However, inflammation is a critical aspect of the recovery process; an excessive and uncontrolled inflammatory response may cause secondary damage to organs (AbuBakr et al. 2018). Systemic release of inflammatory mediators may have profound effects on distant organs. This systemic inflammatory response syndrome (SIRS) may lead to increased vascular permeability, tissue edema, and deteriorated organ function (Greenhalgh 2019; Laggner et al. 2022). The lungs, the liver, and the kidneys are particularly susceptible due to their high vascularization and sensitivity to inflammatory changes (Kotzbeck et al. 2019). Among these, the adrenal glands play an essential role in the body’s reaction to stress and the maintenance of homeostasis.

The adrenal glands, located on top of the kidneys, are endocrine organs responsible for the secretion of hormones that help the body manage stress, regulate the metabolism, and maintain the fluid balance (Cappola et al. 2023). These glands are composed of two separate sections: the adrenal cortex and the adrenal medulla. The cortex produces corticosteroids such as cortisol, which have vital importance for specific metabolic processes (Favero et al. 2021). Meanwhile, the medulla secretes catecholamines such as adrenaline and noradrenaline, which play a role in the fight-or-flight response (Cioccari et al. 2020). Severe burns initiate a complex series of physiological reactions, and the adrenal glands are involved in this process (Senel et al. 2010). Burn injuries trigger the release of a large amount of stress hormones as a part of the body’s attempt to cope with the traumatic event (Laggner et al. 2022). The hypothalamic-pituitary-adrenal (HPA) axis, which is an important pathway that regulates stress, is activated and causes an increase in the production of cortisol and other stress hormones (Kotzbeck et al. 2019). In those with severe burns, the adrenal cortex undergoes significant changes (Williams and Herndon 2017). The constant increase in cortisol levels due to burn-induced stress may lead to irregular immune responses. Although cortisol is needed to suppress inflammation, its chronic elevation may suppress immune function, rendering the body more susceptible to infections (Williams and Herndon 2017). Moreover, excessive cortisol secretion may contribute to metabolic disorders, insulin resistance, and impaired wound healing (Ladhani et al. 2021). In some cases, a prolonged stress reaction may induce adrenal failure, decreasing cortisol production (Aissa et al. 2018). The response of the adrenal medulla to severe burns includes an increase in catecholamines, especially adrenalin and noradrenalin (Laggner et al. 2022). These hormones increase the heart rate, blood pressure, and energy mobilization by triggering the fight-or-flight response. Meanwhile, the long-term elevation of catecholamines may lead to high blood pressure and cardiovascular complications such as a higher risk of myocardial ischemia. The damage caused by severe burns to the adrenal glands may lead to broad consequences for the body’s overall homeostasis. Irregular hormonal secretion and prolonged stress response may exacerbate the inflammatory state, endanger immune function, and impair metabolism. Therefore, medical interventions must target these specific problems. Management strategies may involve administering corticosteroid replacement therapy to fix adrenal failure and support immune function. In addition, the control of the stress response via medication and supportive care may help reduce the unfavorable effects of continuous cortisol and catecholamine secretion or prevent the development of adrenal failure.

Severe burns trigger an important systemic reaction characterized by the release of pro-inflammatory cytokines and immune cell activation (Laggner et al. 2022). Interleukin-6 (IL-6) is a key player in this response as it is rapidly released following a burn injury (Keck et al. 2009). The binding of IL-6 to its receptor triggers the activation of JAK enzymes, which in turn phosphorylate signal transducer and activator of transcription 3 (STAT3) (Zhang et al. 2019a, b). When STAT3 is phosphorylated, dimers form and translocate into the nucleus, where they modulate the transcription of the genes involved in tissue repair and cell survival (Li et al. 2022). The activation of the STAT3 pathway and the release of IL-6 in response to severe burns have multifarious effects on the body’s response to trauma, including inflammation, tissue repair, and regeneration, and immunomodulation (Cho et al. 2004; Burgess et al. 2022; Laggner et al. 2022). However, excessive or irregular IL-6/STAT-3 activation may cause systemic inflammatory reactions, impacting distant organs and potentially contributing to complications such as organ dysfunction or multiple organ failure (Zhang et al. 2019b; Jia et al. 2022). Understanding the molecular response mediated by IL-6/STAT-3 in severe burns would be promising for the development of targeted therapeutic interventions. The modulation of this pathway may potentially help manage inflammation, support tissue repair, and prevent the complications related to excessive immune activation.

Anti-inflammatory treatments are essential in the field of burn treatment since burn injuries provoke a substantial inflammatory response (Boldeanu et al. 2020). Commonly used treatments for inflammation include corticosteroids, nonsteroidal anti-inflammatory drugs (NSAIDs), and biological medications like cytokine inhibitors (Roshangar et al. 2019). Each of these solutions possesses unique advantages and constraints.

Corticosteroids are commonly employed for their potent anti-inflammatory properties, which aid in diminishing both the localized and systemic inflammatory reaction after burns (Markiewicz-Gospodarek et al. 2022). Nevertheless, the utilization of these substances is a subject of debate because of notable drawbacks, including heightened susceptibility to infection, prolonged recovery of wounds, and the possibility of adrenal suppression when used over an extended period (Perantie and Brown 2002).

NSAIDs are commonly prescribed due to their ability to alleviate inflammation and pain caused by burn injuries (Markiewicz-Gospodarek et al. 2022). While NSAIDs are generally less potent than corticosteroids, they are favored due to their lower incidence of severe adverse effects. The primary issue associated with NSAIDs is their detrimental impact on renal function and gastrointestinal well-being. This can provide a significant challenge, especially for patients with extensive burns who are already susceptible to renal failure and other systemic complications (Bindu et al. 2020). Immunomodulators have the ability to regulate the immune response and, hence, decrease inflammation. However, it is essential to note that prolonged use of certain immunomodulators may have detrimental effects on the immune system (Roshangar et al. 2019; Boldeanu et al. 2020). Despite the availability of different therapeutic techniques for treating inflammation in burn injuries, each approach has notable limitations (Roshangar et al. 2019). The presence of these disadvantages emphasizes the necessity for ongoing investigation into more efficient and less risky methods of reducing inflammation, which could enhance results for individuals with burn injuries.

Hormonal secretion from the adrenal glands is regulated by complex signaling pathways involving numerous receptors, including the alpha-2 adrenergic receptors (Cho et al. 2004; Purnell et al. 2004; Nguyen et al. 2017; Lee 2019). These receptors are found on the surface of a variety of cell types, including those found in the adrenocortical gland (Blandizzi 2007). When activated, alpha-2 adrenergic receptors influence the body’s stress response by modulating hormonal release from the adrenal cortex, protecting homeostasis. Dexmedetomidine, which is a highly selective α2-adrenergic receptor agonist, has emerged as a valuable multifaceted agent in modern anesthesia and intensive care management (Lankadeva et al. 2021). Its unique pharmacological profile, which is characterized by sedative, analgesic, anxiolytic, and sympatholytic properties, sets it apart from conventional sedative agents (Nguyen et al. 2017). Moreover, its effects extend beyond the field of anesthesia and sedation. Its neuroprotective, anti-inflammatory, and organ-protective properties have broadened its use in fields such as neurosurgery and sepsis management (Qiu et al. 2018; Bao et al. 2019). Dexmedetomidine was shown to block the release of IL-6 and other pro-inflammatory cytokines, likely by modulating the sympathetic nervous system and reducing the stress response (Minaei and Haghdoost-Yazdi 2019). Besides, its anti-inflammatory qualities may contribute to the suppression of excessive STAT3 activation, preventing the series of events that may lead to tissue damage (Zhang et al. 2019a). This modulation may potentially protect adrenal gland function by reducing cell damage and inflammation.

Severe burns not only cause visible damage to the skin but also result in complex systemic impairments that may profoundly affect specific organs. Among these, the adrenal glands have an essential role in the body’s response to stress and the maintenance of homeostasis. This study aims to explore the IL-6- and STAT3-mediated effects of dexmedetomidine, an alpha-2 adrenergic agonist, on the adrenal gland by examining the relationship between severe scald burns and adrenal gland damage in rats.

## Materials and methods

This experimental study was conducted with the approval of Recep Tayyip Erdoğan (RTE) University Animal Research Ethics Committee.

### Experimental animals and study design

This study was conducted at Recep Tayyip Erdogan University, Faculty of Medicine, Experimental Animal Research Laboratory in accordance with the ARRIVE (Animal Research: Reporting of In Vivo Experiments) guidelines concerning the care and use of experimental animals (du Sert et al. 2020). This study used 3–4-month-old 28 male Sprague-Dawley rats weighing 300 ± 50 g. All rats were kept in an environment with a 12-h light/darkness cycle at a temperature of 22 ± 2°C and a humidity of 55–60%, with free access to food and water.

Twenty-eight rats were randomly distributed to cages of seven. The sample size of the study was calculated in accordance with the studies of Arifin et al (Arifin and Zahiruddin 2017). Randomization was ensured by using a computer-based number generator. Rats in Group 1 (*n*=7, control group) were exposed to 25°C water for 17 s on day 1 and received only 0.09% intraperitoneal (i.p.) NaCl (saline water) is used for 3 days, starting on the same day as water exposure. Rats in Group 2 were exposed to 25°C water for 17 s on day 1 and received 100 mcg/kg/day dexmedetomidine i.p. for 3 days starting the same day as water exposure (Gonullu et al. 2014). For rats in Group 3 (*n*=7, Burn Group), dorsal fur was shaved. On the shaved area, an empty 2-cm cylinder was placed, and boiling water of 94°C was applied inside for 17 s. For rats in Group 4 (*n*=7, Burn+DEX Group), dorsal fur was shaved. Starting from the boiling water application, 100 mcg/kg/day of dexmedetomidine i.p. was administered for 3 days (Gonullu et al. 2014). The burn percentage was set to be 30%. The burn formation model was performed according to the method in the studies of Vorauer-Uhl et al and Ozdemir et al (Vorauer-Uhl et al. 2002; Ozdemir et al. 2023). The surgical procedures and the burn model technique were conducted using anesthesia consisting of 100 mg/kg of ketamine and 10 mg/kg of xylazine under sterile conditions. Rats that had been subjected to burns were administered a single intraperitoneal dose of fentanyl at a concentration of 1.5 mcg/kg once daily for three days to provide pain relief until the termination of the experiment. The rats were sacrificed by decapitation under 100 mg/kg of ketamine and 10 mg/kg of xylazine anesthesia 14 h after the last dexmedetomidine administration (Tsukamoto et al. 2018). The right and left adrenal gland tissues excised from the rats were placed in a 10% neutral formalin solution for histopathological and immunohistochemical analyses.

### Histopathological analysis

The adrenal gland tissue samples were subjected to standard histological preparation protocols, wherein they were immersed in a 10% neutral formalin solution (Sigma Aldrich, St. Louis, MO, USA) for 24 h. After fixation, adrenal gland tissue samples underwent dehydration by being sequentially immersed in a series of increasing concentrations of alcohol (Merck GmbH, Darmstadt, Germany) using a tissue processing device (Shendon Citadel 2000, Thermo Scientific Inc., Waltham, MA, USA). Subsequently, the specimens underwent purification through two successive immersions in xylol solutions obtained from Merck, a reputable manufacturer based in Darmstadt, Germany. The adrenal gland tissues were subjected to embedding in both soft and hard paraffin (Merck GmbH, Darmstadt, Germany) and subsequently embedded into paraffin blocks. Sections of a thickness of 5 µm were obtained by employing a rotary microtome (Leica RM2525, Leica Biosystems, Wetzlar, Germany). The sections were stained with Harris hematoxylin and Eosin G (H&E) (Merck, Darmstadt, Germany) using a histological stainer (Leica Biosystems, 5020ST, Wetzlar, Germany).

### Immunohistochemical (IHC) analysis

The examination of adrenal gland tissue sections was conducted using a TUNEL assay kit (TUNEL Assay Kit - HRP-DAB, ab206386, Abcam, UK), together with primary antibodies for STAT3 (ab68173, Abcam, UK) and IL-6 (ab9324, Abcam, UK). In addition, a secondary antibody (Goat Anti-Rabbit IgG H&L HRP, ab205718, Abcam, UK) was employed with the primary antibody. After the deparaffinization process, the adrenal gland tissue sections, which were 2–3 μm in thickness, underwent treatment with a 3% H2O2 solution for 15 min. This treatment was performed in order to inhibit the activity of endogenous peroxidase, utilizing a Bond MAX IHC/ISH instrument manufactured by Leica Biosystems in Wetzlar, Germany. To prevent background staining, a secondary blocking solution was administered for 20 min. Subsequently, the tissues were subjected to an incubation period of 60 min with the main antibody. Following the administration of the main antibody, the tissue specimens underwent incubation with a secondary antibody. The tissues were treated with a solution of diaminobenzidine chromogen (DAB Chromogen, Abcam, Cambridge, UK), followed by visualization of an image signal using a light microscope. The adrenal gland tissues were ultimately subjected to counterstaining with Harris hematoxylin (Merck, Darmstadt, Germany) and thereafter coated with a suitable solution. To reduce inaccuracies when evaluating the immunopositivity of sections incubated with primary antibodies, negative controls stained solely with H&E (without primary antibodies) were provided.

### Semi-quantitative analysis

Histopathological examination of adrenal gland tissue sections stained with H&E was conducted to assess histopathological damage. The scoring system considered the presence of necrotic cells and hemorrhagic findings. This approach aligns with previous pathological investigations on adrenal gland tissue damage caused by hypovolemic shock, as presented in Table 1 (Rushing et al. 2006). The histologist, who was unaware of the research groups, evaluated a total of twenty distinct regions within each portion. Immunohistochemical techniques were employed to identify the presence of TUNEL, STAT3, and IL-6 positivity in the adrenal gland cells, as presented in Table 2 (Rushing et al. 2006). The histologist, who was unaware of the research groups, assessed a total of twenty distinct regions from each portion of the adrenal gland.

**Table 1:** Histopathological damage score (HDS)

Cortical necrosis	
0	≤5%
1	≤25%
2	≤50%
3	≥51%
Medullar Necrosis	
0	≤5%
1	≤25%
2	≤50%
3	≥51%
Cortical hemorrhage	
0	≤5%
1	≤25%
2	≤50%
3	≥51%
Medullar hemorrhage	
0	≤5%
1	≤25%
2	≤50%
3	≥51%

**Table 2:** Immun-positivity score

Score	Findings
0	≤5%
1	≤25%
2	≤50%
3	≥51%

### Statistical analysis

The statistical tool used to calculate the data received from the analyses was SPSS 20.0 (IBM Corp., Armonk, NJ, USA). The adrenal gland's histopathological damage scoring, immunological positive cell scoring, and semi-quantitative data were found to deviate from a normal distribution based on the results of Shapiro-Wilk, Skewness-Kurtosis, Q-Q Plot, and Levene’s tests. The nonparametric data were represented by the mean and the 25th to 75th percentile. Significant variations were observed across the groups with the Kruskal-Wallis test regarding cortical necrosis, medullary necrosis, cortical hemorrhage, medullary hemorrhage, HDS, TUNEL positivity score, stat3, and IL-6 immunopositivity. Dunn’s tests were used following the Kruskal-Wallis to assess the differences between groups due to the nonparametric and ordinal nature of the data. Statistically significant results were defined as having *P* values less than 0.05.

This is an explorative study conducted in accordance with the EQUIPD guidelines. It does not test a null hypothesis and aims to open the door for research questions that will produce new hypotheses (Vollert et al. 2022). Therefore, the *p*-value calculated in this study should not be interpreted as a hypothesis test but simply as a descriptive value due to the explorative nature of the study.

## Results

### Histopathological results

**Fig. 1:**
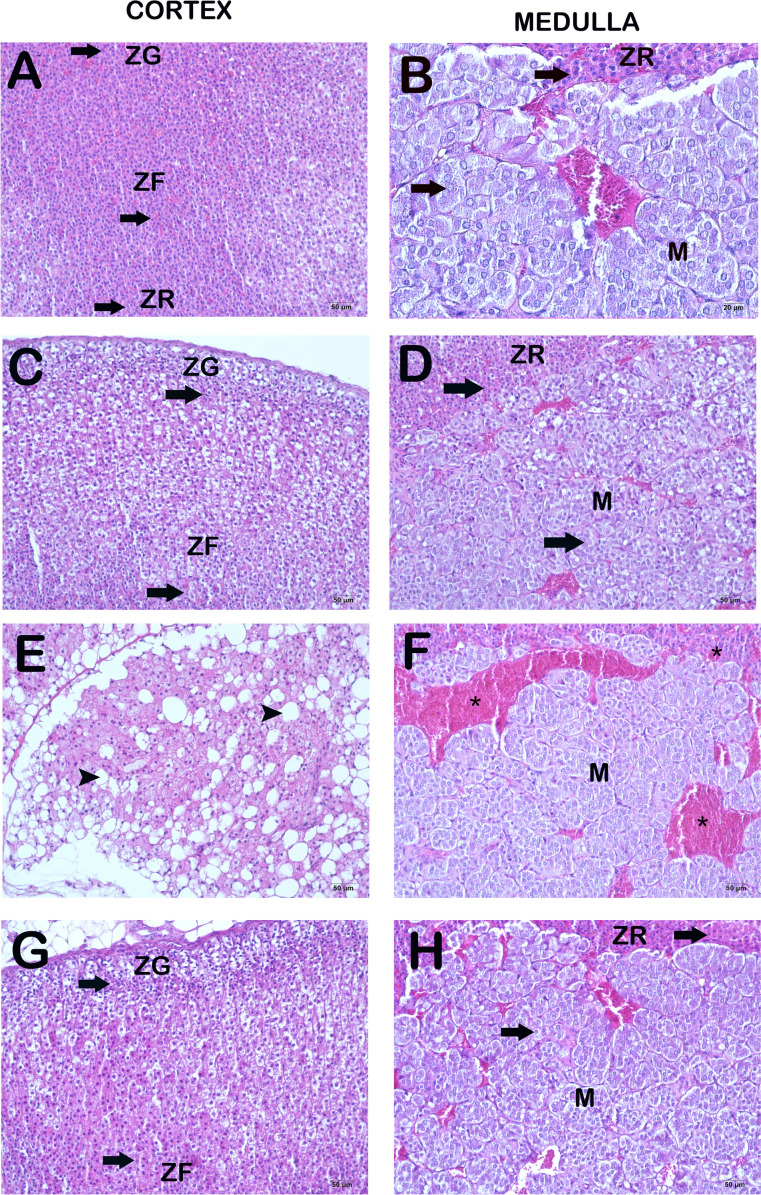
Representative light microscopic images of adrenal gland sections stained with H+E. The Zona Glomerulosa (ZG), Zona fasciculata (ZF), Zona Reticularis (ZR), and Medulla (M) are distinct regions inside the adrenal gland. **A(x20)-B(x20) Control Group:** Normally structured cells are observed in the Zona glomerulosa, Zona fasciculate, and Zona reticularis layers of the adrenal cortex of the control group (arrow). It is observed that the adrenal medulla cells have a normal structure (HDS: 0(0-1). **C(x20)-D(x20) DEX Group:** In the DEX group, it is observed that the Zona glomerulosa, Zona fasciculate, Zona reticularis layers in the adrenal cortex, and the cells in the adrenal medulla have a typical structure (arrow) (HDS): 0(0-1). **E(x20)-F(x20) Burn Group:** In the Burn group, densely necrotic cells (tailed arrow) are observed in the Zona glomerulosa, Zona fasciculata, and Zona reticularis layers of the adrenal cortex. It is observed that there are many necrotic cells in the adrenal medulla region. In addition, widespread hemorrhagic areas are observed in the adrenal cortex and medulla (HDS: 8(7-9). **G(x20)-H(x20) Burn+DEX Group:** In the Burn+DEX group, the number of necrotic cells in the Zona glomerulosa, Zona fasciculata and Zona reticularis layers of the adrenal cortex decreased (arrow). However, it is observed that there is a decrease in the number of necrotic cells and hemorrhagic areas in the adrenal medulla region (HDS: 3(3-4)

Upon examination of the H&E-stained sections under a light microscope, it was seen that the cortical layers of the adrenal gland sections in the control group, namely the zona glomerulosa, zona fasciculate, and zona reticularis, exhibited a typical and unaltered structure. Furthermore, it was noted that the cellular composition in the medulla region of the adrenal gland exhibited a typical structure (Fig. 1a–b, Table 3, HDS: 0(0-1)). In a similar vein, it was noted that the cellular composition of the cortex and medulla regions of the adrenal gland had a characteristic arrangement in the group subjected to dexmedetomidine administration (Fig. 1c–d, Table 3, HDS: 0(0-1)). In contrast, upon microscopic examination of the Burn group sections, a notable presence of necrotic cells was observed in the zona glomerulosa, zona fasciculate, and zona reticularis regions, with a particular emphasis on the cortical area. Furthermore, our investigation revealed the presence of extensive hemorrhagic regions (Fig. 1e–f, Table 3, HDS: 8(7-9)). In contrast, upon microscopic examination of the sections from the Burn+DEX application group, it was observed that the presence of necrotic cells in the zona glomerulosa, zona fasciculate, zona reticularis, and medulla areas exhibited a decrease in comparison to the Burn group. Furthermore, there was a decrease in the hemorrhagic areas (Fig. 1g–h, Table 3, *p*=0.002, HDS: 3(3-4)).

**Table 3:** Histopathological damage score results (HDS, median-25–75% interquartile range)

Group	Cortical Necrosis Score	Medullar Necrosis Score	Cortical Hemorrhage Score	Medullar Hemorrhage Score	HDS
Control	0(0-0)	0(0-0)	0(0-0)	0(0-0)	0(0-1)
DEX	0(0-0)	0(0-0)	0(0-0)	0(0-0)	0(0-1)
Burn	2(2-3)^a,b^	2(2-2)^a,b^	2(2-2)^a,b^	1(1-2)^a,b^	8(7-9)^a,b^
Burn+DEX	1(1-1)^a,b,c^	1(0-1)^a,b,c^	1(0-1)^a,b,c^	1(0-1)^d,e,f^	3(3-4)^a,b,g^

^a^*p<*0.001 compared to Control Group,

^b^*p<*0.001 compared to DEX Group,

^c^*p<*0.001 compared to Burn Group,

^d^*p*=0.001 compared to Control group

^e^*p*=0.029 compared to DEX Group

^f^*p*=0.003 compared to Burn Group

^g^*p*=0.002 compared to Burn group

Post hoc Dunn’s Test following Kruskal-Wallis test

### Immunohistochemical results

#### TUNEL positivity

**Fig. 2:**
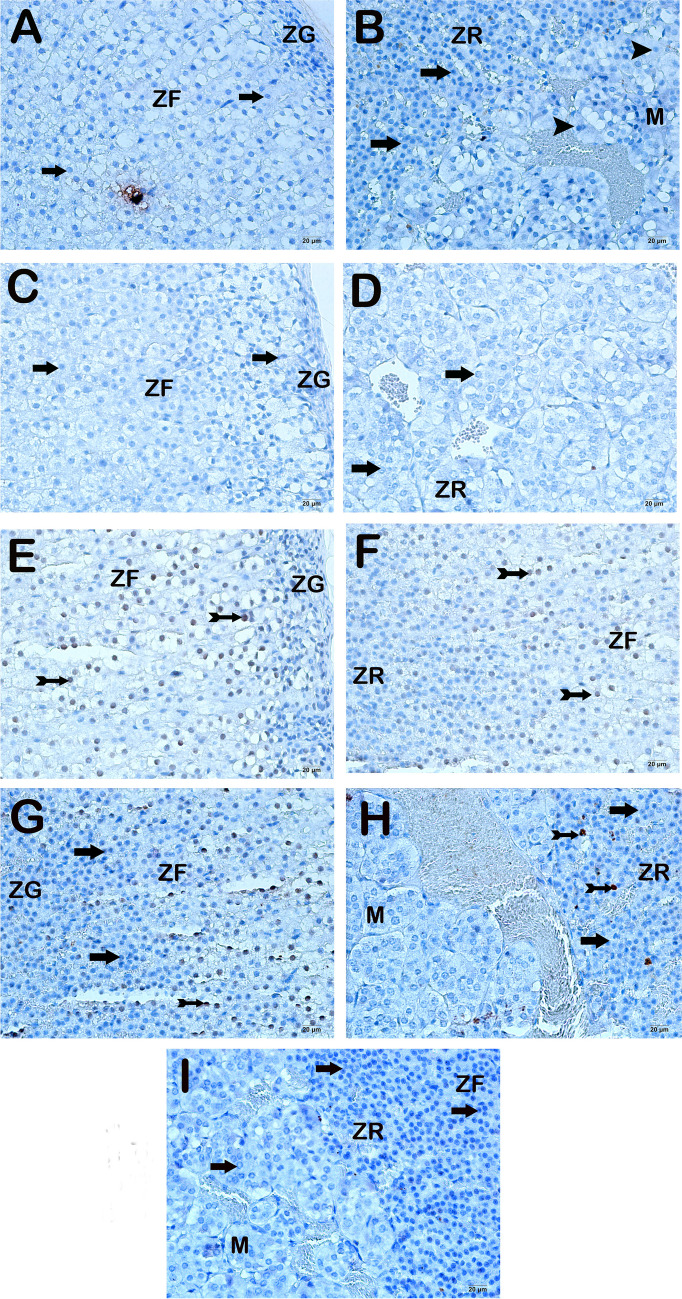
Representative light microscopic images of sections of the adrenal gland stained with the TUNEL method. Zona Glomerulosa (ZG), Zona Fasciculata (ZF), Zona Reticularis (ZR), Medulla (M). **A(x20)-B(x20) Control Group:** It is observed that the zona glomerulosa, zona fasciculata and zona reticularis cells in the adrenal cortex region have a normal structure and are immune-negative (arrow). In addition, TUNEL-negative cells are observed in the adrenal medulla region (TUNEL positivity score 0 (0-0)). **C(x20)-D(x20) DEX Group:** It is observed that the zona glomerulosa, zona fasciculata and zona reticularis cells in the adrenal cortex region have a typical structure and are immune-negative (arrow, TUNEL positivity score 0 (0-0)). **E(x20)-F(x20) Burn Group:** Apoptotic cells showing intense TUNEL-positivity are observed in the zona glomerulosa, zona fasciculata, zona reticularis and medulla regions (tailed arrow, TUNEL positivity score 2(2-2)). **G(x20)-H(x20) Burn+DEX Group:** It is observed that apoptotic cells (tailed arrow) showing TUNEL positivity have decreased in the Zona glomerulosa, zona fasciculata, zona reticularis of the adrenal cortex and adrenal medulla regions (TUNEL positivity score 1(1-1)). **I(x20) Negative Control:** TUNEL-negative cells (arrow) are observed in the adrenal medulla region

In our study, it was revealed that within the control group, specific regions of the rat adrenal gland, including the zona glomerulosa, zona fasciculate, zona reticularis, and medulla, included immune-negative cells exhibiting characteristic structures (Fig. 2a–b, Table 4, TUNEL positive score: 0(0-0)). In a similar vein, it was noted that immune-negative cells exhibiting characteristic structures were present in both the cortex and medulla areas of the adrenal gland in the sections from the DEX group (Fig. 2c–d, Table 4, TUNEL positivity score: 0(0-0)). In contrast, our analysis of the Burn group revealed a notable presence of high TUNEL positivity in several cells, particularly within the zona glomerulosa, zona fasciculate, zona reticularis, and medulla areas (Fig. 2e–f, Table 4, TUNEL positivity score: 2(2-2)). A decrease in the frequency of apoptotic cells exhibiting high TUNEL positivity was found in the zona glomerulosa, zona fasciculata, zona reticularis, and medulla areas of the Burn+DEX group compared to the Burn group (Fig. 2g–h, Table 4, *p*<0.001, TUNEL positivity score: 1(1-1)).

**Table 4:** Immune-positivity score results (HDS, median-25–75% interquartile range)

Group	TUNEL Positivity Score	STAT3 Positivity Score	IL-6 Positivity Score
Control	0(0-0)	0(0-0)	0(0-0)
DEX	0(0-0)	0(0-0)	0(0-0)
Burn	2(2-2)^a,b^	2.5(2-3)^a,b^	2(2-2)^a,b^
Burn+DEX	1(1-1)^a,b,c^	1(1-1)^c,d,e^	0(0-1)^f^

^a^*p<*0.001 compared to Control Group,

^b^*p<*0.001 compared to DEX Group,

^c^*p*=0.001 compared to Burn Group,

^d^*p*=0.001 compared to Control Group

^e^*p*=0.002 compared to DEX Group

^f^*p*<0.001 compared to Burn Group

Post hoc Dunn’s Test following Kruskal-Wallis test

#### STAT3 positivity

**Fig. 3:**
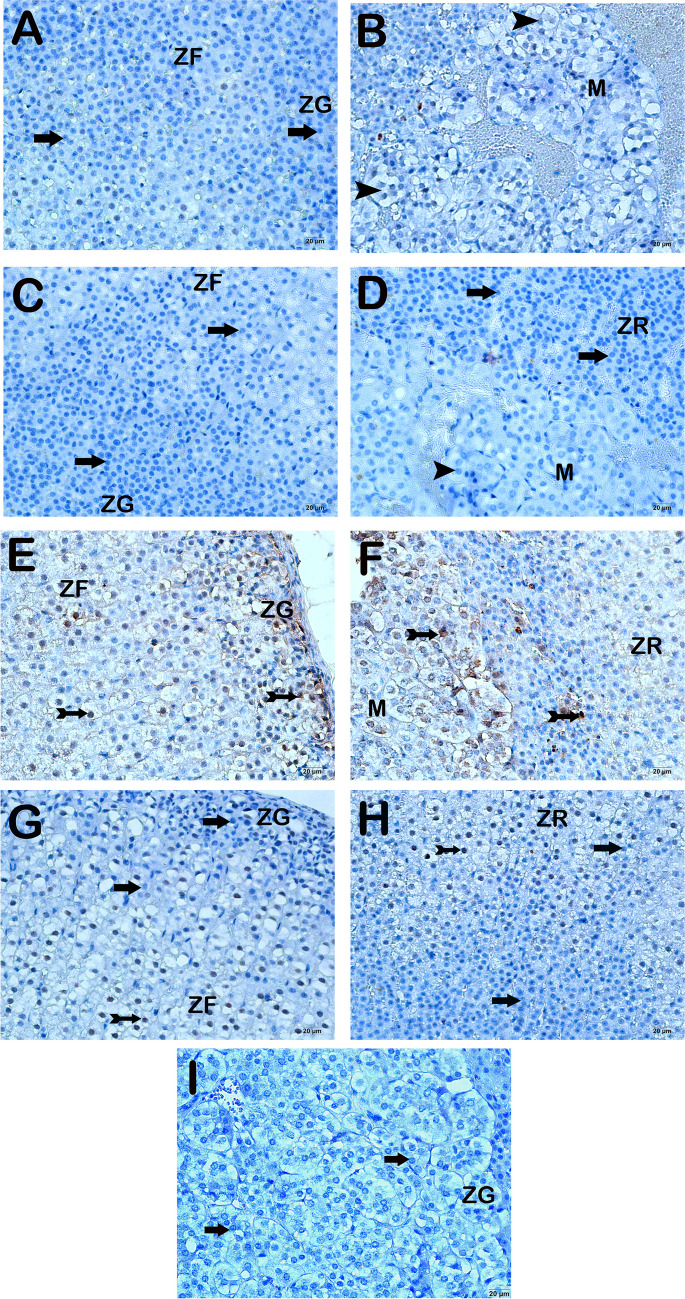
Representative light microscopic images of sections of the adrenal gland stained with STAT3 primary antibody. Zona Glomerulosa (ZG), Zona Fasciculata (ZF), Zona Reticularis (ZR), Medulla (M).** A(x20)-B(x20) Control Group:** It is observed that the cells in the zona glomerulosa, zona fasciculata, and zona reticularis layers in the adrenal cortex region are STAT3 negative. In addition, STAT3 negative cells are observed in the medulla region (arrow, STAT3 positivity score 0 (0-0)). **C(x20)-D(x20) DEX Group:** It is observed that the cells in the zona glomerulosa, zona fasciculata, and zona reticularis layers of the adrenal cortex have a typical structure and are STAT3 negative (arrow, STAT3 positivity score 0 (0-0)). **E(x20)-F(x20) Burn Group:** It is observed that the cells in the zona glomerulosa, zona fasciculata, zona reticularis, and medulla region show intense STAT3 positivity (tailed arrow, STAT3 positivity score 2.5(2-3)). **G(x20)-H(x20) Burn+DEX Group:** It is observed that STAT3-positive cells (tailed arrow) are decreased in the Zona glomerulosa, zona fasciculata, and zona reticularis and adrenal medulla regions (STAT3 positivity score 1(1-1)). **I(x20) Negative Control:** STAT3-negative cells (arrow) are observed in the adrenal medulla region

Upon examination of sections of adrenal gland tissue incubated with a primary antibody for STAT3 under a light microscope, it was observed that there were cells lacking STAT3 immune reactivity in the zona glomerulosa, zona fasciculate, zona reticularis, and medulla regions in both the control and DEX groups (Fig. 3a–d, Table 4, STAT3 positivity score: 0(0-0). ); 0(0-0)). In contrast, the adrenal gland tissue sections from the Burn group had a notable presence of cells with extensive STAT3 positivity in the zona glomerulosa, zona fasciculata, zona reticularis, and medulla regions compared to the control and DEX groups (Fig. 3e–f, Table 4, *p*<0.001, *p*<0.001, respectively, STAT3 positivity score: 2(2-2)). In contrast, our findings indicate a decrease in the presence of cells expressing STAT3 in both the adrenal cortex and medulla of the Burn+DEX application group sections, as compared to the Burn group (Fig. 3g–h, Table 4, *p*<0.001; STAT3 positive score: 1(1-1)).

#### IL-6 positivity

**Fig. 4:**
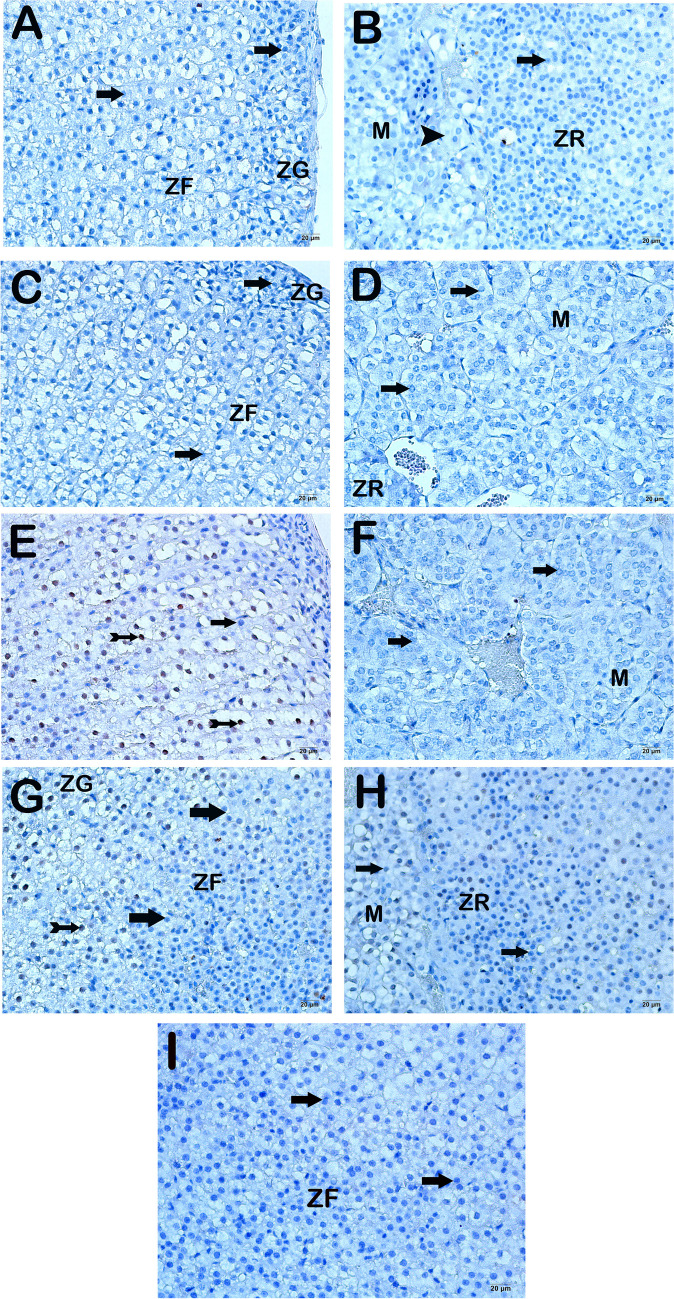
Representative light microscopic images of sections of the adrenal gland stained with IL-6 primary antibody. Zona Glomerulosa (ZG), Zona Fasciculata (ZF), Zona Reticularis (ZR), Medulla (M).** A(x20)-B(x20) Control Group:** It is observed that the cells in the zona glomerulosa, zona fasciculata, and zona reticularis layers in the adrenal cortex region are IL-6 negative (arrow). In addition, immune-negative cells are observed in the medulla region (IL-6 positivity score 0 (0-0)). **C(x20)-D(x20) DEX Group:** It is observed that the zona glomerulosa, zona fasciculata and zona reticularis cells in the adrenal cortex region have a typical structure and are immune-negative (arrow, IL-6 positivity score 0 (0-0)). **E(x20)-F(x20) Burn Group:** It is observed that the cells in the zona glomerulosa, zona fasciculate, zona reticularis, and medulla region show intense IL-6 positivity (tailed arrow, IL-6 positivity score 2(2-)). **G(x20)-H(x20) Burn+DEX Group:** It is observed that the cells showing intense IL-6 positivity in the zona glomerulosa, zona fasciculata, zona reticularis, and medulla regions have decreased (tailed arrow, IL-6 positivity score 0 (0-1)). **I(x20) Negative Control:** IL-6-negative cells (arrow) are observed in the adrenal medulla region

Upon examination of adrenal gland tissue sections incubated with an IL-6 primary antibody using a light microscope, it was observed that the control group exhibited immune-negative cells with a characteristic structure in the zona glomerulosa, zona fasciculata, zona reticularis, and medulla regions (Fig. 4a–b, Table 4, IL-6 positivity score: 0(0). -0)). Likewise, within the DEX group, the adrenal cortex and medulla regions exhibited immune-negative cells expressing IL-6 (Fig. 4c–d, Table 4, IL-6 positivity score: 0(0-0)). In contrast, the adrenal gland tissue sections from the Burn group had a notable presence of cells with extensive IL-6 positivity throughout many locations, including the zona glomerulosa, zona fasciculate, zona reticularis, and medulla, compared to the control and DEX groups (Fig. 4e–f, Table 4, *p*<0.001, *p*<0.001, respectively, IL-6 positivity score: 2(2-2)). In contrast, our findings indicate a decrease in the presence of cells expressing IL-6 in both the adrenal cortex and medulla of the sections from the Burn+DEX treatment group, as compared to the Burn group (Fig. 4g–h, Table 4, *p*<0.001; IL-6 positive score: 0(0-1)).

## Discussion

Scald burns constitute a type of thermal injury that may lead to systemic inflammation and multiple organ failure (Jeschke et al. 2020). Among the affected organs, the adrenal glands play a critical role in the body’s response to stress and inflammation(Kotzbeck et al. 2019). This study investigated the potential role of dexmedetomidine in modulating the interaction between the pro-inflammatory cytokine IL-6 and the STAT3 pathway in adrenal gland injury that was indirectly induced by scald burns in rats. Several studies have demonstrated the anti-inflammatory properties of dexmedetomidine (Lankadeva et al. 2021; Lee et al. 2021). This α2-adrenergic receptor agonist was shown to suppress the release of pro-inflammatory cytokines, including IL-6 in various experimental models (Liu et al. 2022). In line with these findings, our results show a significant decrease in IL-6 levels in the dexmedetomidine-treated groups compared to the control group. Dexmedetomidine inhibits the release of IL-6 and other pro-inflammatory cytokines, likely by modulating the sympathetic nervous system and reducing the stress response (Wang et al. 2022). This suggests that dexmedetomidine may alleviate the initial inflammatory response triggered by scald burns.

Interleukin-6 is a pleiotropic cytokine that plays a dual role as both a pro-inflammatory and anti-inflammatory mediator in acute as well as chronic inflammation (Meng et al. 2018). It is known to be upregulated in response to various types of injury, including thermal burns (Costantini et al. 2022). It exerts its effects by binding to its receptor and leads to the activation of downstream signaling pathways, including Janus kinase (JAK) and STAT3 (Alten et al. 2008). STAT3 activation mediates various cellular responses, including cell survival, reproduction, and inflammation (Zhang et al. 2019a). The STAT3 signaling pathway is a critical regulator of inflammatory and immune responses (Chen et al. 2017). However, excessive or prolonged activation of this pathway may contribute to tissue damage and pathological conditions (Li et al. 2022). In the present study, we observed an increase in STAT3 activation in response to scald burns. Interestingly, dexmedetomidine treatment was found to decrease STAT3 activation. Given that permanent STAT3 activation is associated with long-term inflammation and tissue damage, this may have considerable consequences. The anti-inflammatory properties of dexmedetomidine can reduce the production of pro-inflammatory mediators and prevent the series of events leading to tissue damage by contributing to the suppression of excessive STAT3 activation.

Dexmedetomidine, which is a highly selective α2-adrenergic agonist, has received attention due to its potential anti-inflammatory and organ-protective qualities (Pichot et al. 2010; Mantz et al. 2011). Its effects on the modulation of inflammatory pathways, including the JAK-STAT pathway, have been investigated in various studies (Si et al. 2013, 2014; Jia et al. 2022). Particularly, it was shown to suppress STAT3 activation in a variety of inflammation and injury models, including neuroinflammation, intestinal mucosal injury induced by ischemia-reperfusion damage, as well as heart, kidney, lung, and liver injury induced by sepsis and ischemia-reperfusion (Si et al. 2013, 2014; Chen et al. 2017, 2023; Zhang et al. 2019a; Pan et al. 2020; Jia et al. 2022; Li et al. 2022; Liu et al. 2022). Its ability to reduce IL-6 expression and inhibit STAT3 activation may contribute to its anti-inflammatory and organ-protective qualities. By suppressing these steps of pro-inflammatory signaling, dexmedetomidine may alleviate the inflammatory response in the adrenal glands, protecting their function and preventing further damage. Moreover, dexmedetomidine’s potential to modulate apoptosis and oxidative stress may have a synergic effect on the IL-6/STAT3 pathway. Studies show that STAT3 activation may trigger anti-apoptotic responses (Chen et al. 2017; Li et al. 2022). The reduced TUNEL immunopositivity in the adrenal glands of rats treated with dexmedetomidine in our study supports the ability of dexmedetomidine to reduce apoptosis. Dexmedetomidine’s ability to support anti-apoptotic factors may further reduce adrenal cell damage (Zhang et al. 2021).

The adrenal gland plays a vital role in the body’s response to stress and injury (Venn et al. 2001). However, it is quite susceptible to damage during severe burns due to the release of stress hormones and inflammatory mediators (AbuBakr et al. 2018). Our study indicates that dexmedetomidine treatment reduces the histopathological changes in the adrenal gland induced by scald burns. This suggests that dexmedetomidine may exert a protective effect on this vital organ.

Alpha-2 adrenergic receptors belong to the G-protein coupled receptors (GPCRs) family and are found on the surface of various cells, including the cells of the adrenocortical gland (Lee 2019; Lankadeva et al. 2021). The complex relationship between alpha-2 adrenergic receptors and the adrenocortical gland highlights the complex mechanisms that govern the hormonal balance of our body. The activation of these receptors by stress-related neurotransmitters provides a unique perspective on how finely the body’s response to stress is tuned to prevent the overactivity of stress hormones. These receptors are activated by norepinephrine and epinephrine, which are neurotransmitters produced by the sympathetic nervous system (Miksa et al. 2009). The activation of alpha-2 adrenergic receptors has variable effects depending on their physical location (Lee 2019).

The interaction between alpha-2 adrenergic receptors and the adrenocortical gland is particularly important during the body’s response to stress. In the context of the adrenocortical gland, the activation of alpha-2 adrenergic receptors may lead to the inhibition of hormonal secretion (Venn et al. 2001). Stress triggers norepinephrine and epinephrine release by activating alpha-2 adrenergic receptors in the adrenal cortex (Aissa et al. 2018). When norepinephrine or epinephrine binds to these receptors, a signal chain that ultimately reduces the activity of the enzymes responsible for the synthesis of cortisol and other steroids is initiated (Wang et al. 2022). This negative feedback loop helps prevent excessive hormonal secretion and contributes to the fine-tuned regulation of hormonal levels. This activation plays a critical role in the reduction of cortisol release, which is generally described as the “stress hormone” (Gu et al. 2015). By reducing cortisol overproduction, alpha-2 adrenergic receptors help prevent an excessive stress response, which may be harmful to the body. Accordingly, dexmedetomidine is known to reduce the stress response related to surgery and decrease the plasma concentrations of catecholamines (Si et al. 2014).

Drugs that target the alpha-2 adrenergic receptors, which are known as alpha-2 agonists, are used to treat conditions like hypertension, anxiety, and pain (Nguyen et al. 2017). Through the modulation of these receptors, these drugs may affect hormonal secretion in the adrenal cortex, indirectly influencing processes such as the regulation of blood pressure and the stress response (Lankadeva et al. 2021). Moreover, the irregularity of alpha-2 adrenergic receptor function in the adrenocortical gland has been demonstrated in certain medical conditions (González-Gil et al. 2015). For example, disorders such as Cushing syndrome, which is characterized by excessive cortisol production, may be caused by defective alpha-2 adrenergic receptors that lead to poor inhibition of cortisol synthesis (Fleseriu et al. 2021).

As a concern regarding dexmedetomidine’s relationship with adrenal failure, the literature contains a case report of a 1-year-old child who presented with a second-degree burn and developed temporary adrenal failure, which was thought to be induced by dexmedetomidine infusion (Tucker et al. 2013). However, in this case, which was limited to a single patient, it is possible that the cause for the development of adrenal failure was associated with a critical disease or the other sedative drugs that were used. Because in clinical studies that included a greater number of surgical intensive care patients, dexmedetomidine did not cause adrenal failure and was even shown to weaken the inhibition of etomidate on adrenocortical function in elderly patients as well as protect intraoperative hemodynamic stability (Wang et al. 2022). Moreover, another study showed that dexmedetomidine decreased IL-6 levels and did not inhibit adrenal steroidogenesis in postoperative intensive care patients (Venn et al. 2001). Upon consideration of the results of these studies and our study in combination, we think that dexmedetomidine can provide protection against hypercortisolism by inhibiting the excessive stress response without causing adrenal failure.

According to the result of our review of the literature, the present study is the first pilot study that has investigated the effects of dexmedetomidine on burn-induced secondary adrenal damage via the IL-6 and STAT3 pathways using a rat model. Certain limitations of our study should be taken into consideration. Firstly, this is an animal model study. Therefore, it cannot be applied to clinical practice before conducting human experiments. Also, the results of this study were evaluated in the acute period. Long-term studies are needed for the long-term clinical results. Our study did not include biochemical and hormonal analyses. On the other hand, our study revealed a new mechanism of dexmedetomidine in reducing burn-induced histopathological damage in adrenal glands. The IL-6/STAT3 pathway may be a new therapeutic target in the suppression of excessive inflammation occurring after severe burns.

## Conclusion

The results of this study suggest that dexmedetomidine may have a therapeutic potential in the treatment of scald burn injuries. By decreasing IL-6 secretion and modulating the STAT3 pathway, dexmedetomidine may help alleviate the systemic inflammatory response that is often associated with severe burns. Moreover, the protective effects of dexmedetomidine on the adrenal gland may contribute to the improvement of the outcomes in burn patients. However, it is crucial to consider that the complexity of the inflammatory response in burn patients cannot be attributed solely to the IL-6 and STAT3 pathways. Other signaling pathways, cytokines, and immune cells also play essential roles in the response to burn injuries. Additionally, the results of our study should be confirmed by different methods such as ELISA and Western Blot. Future studies should investigate the broader immunomodulatory effects of dexmedetomidine, as well as the optimal dose and time of application in burn injury models. In addition, understanding the role of alpha-2 adrenergic receptors in the adrenal cortex will light the way for potential therapeutic interventions for conditions related to irregular cortisol. Disorders such as Cushing syndrome, characterized by cortisol overproduction, and Addison’s disease, characterized by insufficient cortisol production, may benefit from drugs that will selectively modulate the alpha-2 adrenergic receptors in the adrenal cortex.

In conclusion, our study presents evidence indicating that dexmedetomidine may reduce scald burn-induced adrenal gland damage by regulating the IL-6 and STAT3 pathways. These results have potential clinical implications for the treatment of burn injuries; however, more studies are necessary to completely elucidate the underlying mechanisms and evaluate the translation of these results to human patients.

## Data Availability

All data generated or analyzed during this study are included in this article. Further inquiries can be directed to the corresponding author.
